# Case Report: Unexplained Fever and Chest Pain in a 5-Year-Old

**DOI:** 10.3389/fped.2021.694678

**Published:** 2021-06-22

**Authors:** Paulina Lubocka, Robert Sabiniewicz

**Affiliations:** Department of Pediatric Cardiology and Congenital Heart Disease, Medical University of Gdańsk, Gdańsk, Poland

**Keywords:** case report, chest pain, pericarditis, prednisone, thoracic injuries

## Abstract

Pericarditis is a rare, but severe cause of chest pain in children that can easily be overlooked during routine diagnostics. Fibrinous pericarditis was recognized in a 5-year old patient who presented with fever and chest pain. Despite thorough diagnostics, no evident etiological factor was found. Furthermore, the disease was unresponsive to broad-spectrum antibiotics and NSAIDs, however oral prednisone was found to cause rapid improvement in the patients' condition. It was presumed that the patient's condition was caused by a blunt trauma to the chest experienced 3 days prior to the onset of symptoms. In an 8-month follow-up the patient remains in good overall condition and no recurrences were observed.

## Introduction

Respiratory tract infections in children are frequently accompanied by chest pain. This symptom, although potentially causing deep concern for parents, is rarely underlined by cardiac disease (1). The patient's ailments result from irritation of sensory nerve endings in the chest wall during intense coughing, remaining in a forced position, viral replication in muscle cells, or a direct inflammation of the pleura. Symptoms may appear at the onset of the inflammatory process as well as in the recovery phase, and may continue for several days following infection. During this time of a global pandemic, when any case of unexplained fever raises suspicions of COVID-19 disease, we present a patient who, despite negative SARS-CoV-2 PCR test, developed a rare condition with similar symptoms.

### Case Description

A 5-year-old female was admitted to the department of pediatrics with a fever of 39.0°C, fatigue and chest pain. The symptoms appeared 3 days prior and were not preceded by an infection. The patient otherwise appeared healthy and did not require any specialist medical care. A SARS-CoV-2 infection was excluded by PCR test, that was negative for both the patient and her mother.

**Figure 1:**
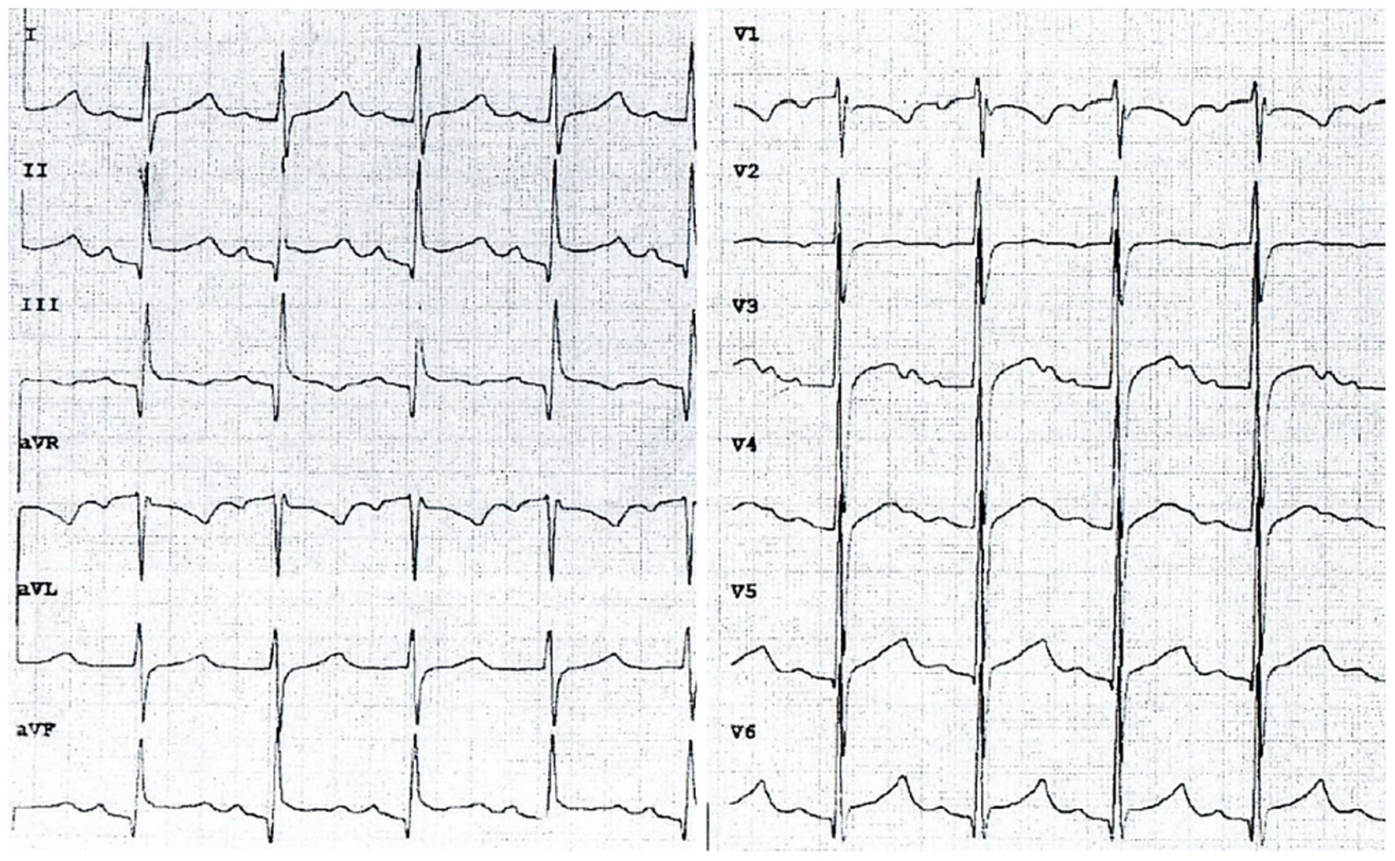
An electrocardiogram on admission to the department of pediatrics showing typical features of acute pericarditis (chart speed: 50 mm/s, calibration: 1 mm/mV). Sinus tachycardia (145 bpm), diffuse concave ST-segment elevation with concomitant PR depression.

Because of tachycardia (150 bpm) and a silent murmur over the heart, echocardiography was ordered, which revealed a thin layer of fluid in the pericardial sac (3.8 mm thick). Additionally, there was a sinus tachycardia with diffuse ST elevations in precordial and limb leads (Figure 1). Laboratory evaluation revealed elevated C-reactive protein (CRP) level of 90 mg/ml (0–5 mg/ml normal range), while troponin (TnI), procalcitonin and complete blood count results within normal range. The tests for borreliosis, tuberculosis and HIV infection were negative as well as blood cultures for multiple organisms. The results of complex autoimmune diagnostics, including anti-nuclear antibodies (ANA), anti-neutrophil cytoplasmic antibodies (ANCA) and anti-Scl-70 antibodies were unremarkable. Since the fever continued, with no satisfactory response to oral paracetamol and ibuprofen, empirical broad-spectrum antibiotic therapy was started. In the following days, as the changes on electrocardiogram disappeared and the layer of pericardial effusion appeared stable, the daily dose of ibuprofen was reduced. Despite that, the patient remained dysphoric and sleepy, with continuous low-grade fever. On her 15th day of admission to hospital, CRP increased to 115 mg/ml, TnI raised to 7 ng/L (0–2.5 ng/L normal range) and echocardiography revealed fibrinous adhesions between the pericardial laminae. High dose ibuprofen was re-administered and continued for another 9 days with the addition of colchicine. On the 24th day, the patient was discharged home with CRP reduced to 25 mg/mL and 2.2 mm thick layer of fluid in the pericardial sac. Ibuprofen (2 × 100 mg per os) and colchicine (1 × 0.5 mg per os) were prescribed until the next out-patient review.

The girl and her mother presented 10 days later at the Emergency Department in a different town with relapse of fever (38.6°C). Once again, the PCR test for SARS-CoV-2 RNA was found to be negative. Considering the patient's past medical history, current symptoms and elevated inflammatory markers, she was transferred to the Clinic of Pediatric Cardiology.

**Figure 2:**
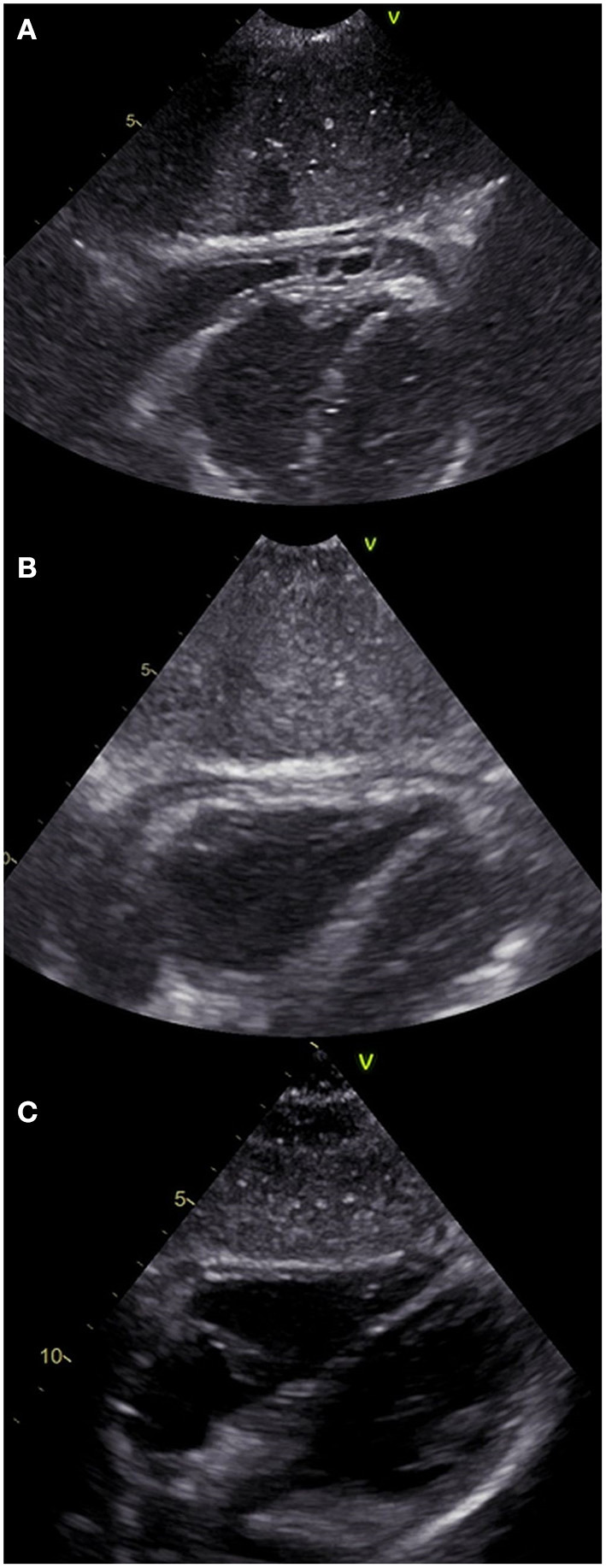
Echocardiographic subcostal view of the patient at different stages of the disease. **(A)** On admission to the Clinic of Pediatric Cardiology (2nd episode of fever). Both of the pericardial laminae are thickened with a pathological layer of fluid in between and fibrous adhesions in the apical region. **(B)** 7th day of hospitalization (4th day on prednisone). The laminae are still thickened, despite considerable reduction of the effusion. **(C)** 7 weeks after discharge showing normal physiological image.

**Figure 3:**
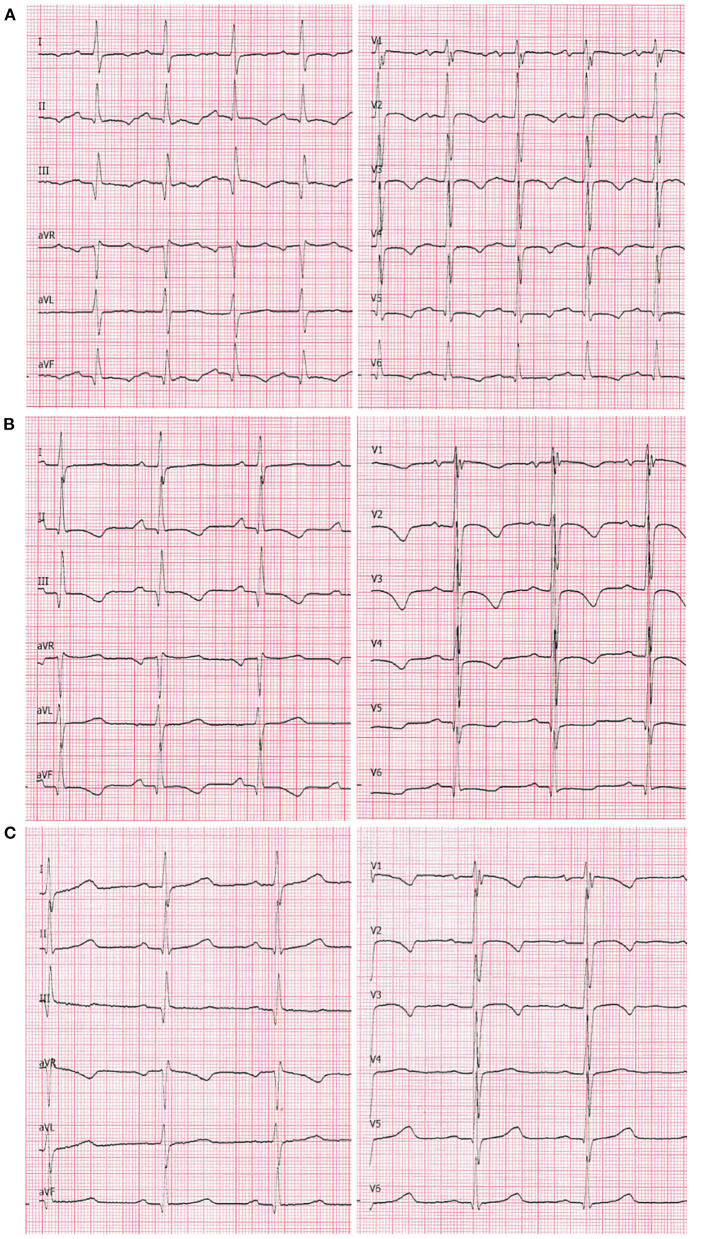
A 12-lead ECG at chart speed of 50 mm/s and calibration of 1 mm/mV. **(A)** On admission to the Clinic of Pediatric Cardiology (2nd episode of fever). Sinus tachycardia (140 bpm), negative T-waves in leads: I, II, III, aVF, and all precordial leads. Even though the QRS voltages are normal for age, they are markedly lower compared with the two following recordings. **(B)** 7th day of hospitalization (4th day on prednisone): sinus rhythm 100 bpm. The negative T-waves are still present in leads II, III, aVF as well as V1-V4; flattened in leads V5 and V6. **(C)** 7 weeks after the discharge: sinus rhythm 81 bpm. The T-waves are negative in leads V1–V3 (adequate for age).

An echocardiogram was performed, which revealed a 4.5 mm thick layer of fluid between pericardial laminae. Moreover, a thickened parietal lamina was connected to the visceral lamina with hyper-echogenic fibrous adhesions (Figure 2A). At the time of examination, the girl did not complain of chest pain or dyspnea. On auscultation, there was a pericardial friction rub over Erb's point and inverted T-waves were recorded on the ECG (Figure 3A). The medical interview taken at that point revealed some additional information. Three days prior to the first episode of fever, the girl underwent a significant blunt force trauma to the chest while playing outside. Initially, she reported superficial pain of her ribs and thoracic spine. She attended the family doctor, who did not find any musculoskeletal lesions. Two days later, the fever appeared, and the location of the pain changed to deep inside the chest.

Considering all the above, the patient was diagnosed with incessant pericarditis and administered ibuprofen (3 × 200 mg daily). The girl was visited by specialists in pediatric rheumatology, hematology, and immunology, however the etiology of her pericarditis remained unknown. Chest CT showed no pathological features over the lungs or mediastinum, and no additional abnormalities in laboratory tests appeared.

After 4 days of treatment, the girls' overall condition improved considerably, however fibrinous pericardial effusion persisted, as did the elevated CRP. Steroid therapy with oral prednisone 1 mg/kg daily was initiated, which improved her condition considerably. On the 7th day of hospitalization (after four doses of prednisone), not only did the CRP level decrease, but the ECG improved (Figure 3B) and the effusion resolved completely (Figure 2B). The patient was discharged home and after 2 weeks of therapy (prednisone, high dose ibuprofen and colchicine) with a satisfactory echocardiographic and clinical effect, the dose of ibuprofen and prednisone were gradually reduced. After 4 weeks, the patient was only receiving colchicine, which was continued for another month. In a 5-month follow-up, she remained asymptomatic, with physiological amounts of pericardial fluid (Figure 2C) and a normal ECG for her age (Figure 3C).

Considering absence of other factors likely to cause pericarditis as well as an immediate response to steroids, we presume that a post-traumatic inflammatory reaction was the most probable etiological mechanism for patients' condition.

## Discussion

Pericarditis is responsible for approximately 5% of chest pain cases in children that require medical consultation. The diagnosis is based on the presence of at least two out of four criteria (1) that include:

- Pericarditic (stabbing) chest pain,
- Pericardial effusion on transthoracic echocardiography,
- Pericardial friction rub,
- Widespread concave ST elevation and PR depression throughout most of the limb leads and precordial leads

At the emergency department our patient fulfilled three of the four criteria.

According to the etiology, pericarditis can be classified as infectious and non-infectious (2–4), with a range of different causes in children (Table 1). Furthermore, there are two major complications of pericarditis. Massive effusion can result in cardiac tamponade, whereas a dry form, with formation of fibrotic adhesions might lead to constriction and diastolic heart failure (5).

**Table 1:** Etiology of pericarditis.

INFECTIVE	viral	*Enteroviridae, Adenoviridae, Herpesviridae*
	bacterial	mainly *M. tuberculosis*, cutaneous bacteria after cardiac surgery, *S. aureus* (blood-borne infection)
	fungal	rare
	paracytic	rare
NON-INFECTIVE	autoimmune	SLE, Sjogren syndrome, RA, JIA, vasculitis, sarcoidosis, FMF
	metabolic	hypothyroidism, uremia, anorexia
	cardiac	chronic heart failure, hypertension, aortic dissection, myocarditis
	mechanical	direct or indirect chest trauma, post-pericardiotomy, post-myocardial infarction, post-ablation
	neoplastic	lymphoma, methastasis, rarely a primary neoplasm (*mesothelioma*)
	drug-induced	cytostatics

*SLE, systemic lupus erythematosus; RA, rheumatoid arthritis; JIA, juvenile idiopathic arthritis; FMF, familial Mediterranean fever*.

Chest pain is the most common complaint reported by pediatric patients with pericarditis. It is typically stabbing, located beneath the sternum and aggravates in a recumbent position. Compared with adults, children with pericarditis frequently suffer from high fever, tachycardia and importantly an increase of inflammatory markers. Less prevalent symptoms include a dry cough, myalgia, arthralgia and fatigue. Pericardial friction rub is a relatively rare (~ 20% of patients) and variable phenomenon (2).

Classically, a series of consecutive changes can be observed on the electrocardiogram (6). In the acute phase, there are ST elevations in multiple leads, that later transform into the T-wave inversions. Additionally, sinus tachycardia and reduced QRS voltage can be seen. Some of these features may persist for weeks or even months after recovery. In the case of the patient described above, information about ST elevations on her ECG could be found in her initial medical documentation, however when she presented to our unit they were no longer detectable. We present the trajectory of her ECG curve from admission to our clinic, to the complete resolution of changes (Figures 3A–C).

Turning to the clinical picture of pericarditis, neither the symptoms nor the echocardiographic picture specific of any etiology. Complications including cardiac tamponade and massive, sometimes irreversible fibrosis, are more frequently observed in bacterial infections (7) and tuberculosis (8) compared to viral pericarditis. However, if treated properly, bacterial pericarditis is characterized by a lower rate of recurrences. In autoimmune conditions, both the dry and exudative pericarditis can be observed, however the heart is rarely the first organ to be affected (9, 10). This patient therefore, presents a range of symptoms characteristic of their principal disease.

The case presented above illustrates a pericarditis of an unclear etiology. Despite a clear coincidence in time between the trauma and following malady, so far no similar cases regarding pediatric patients have been described. Among the mechanical causes of pericarditis, iatrogenic injury is mostly observed. However, among adults a pericardial effusion, fibrosis and tamponade following a blunt force chest injury have also been reported (11, 12). It is uncertain, whether an extremely unfortunate fall can lead to pericarditis without any skeletal lesions. Even though an underlying autoimmune condition cannot be completely excluded in this case (13), in the absence of other clinical or laboratory findings we suggest the post-traumatic etiology as the most probable mechanism. The pathophysiological mechanism of an inflammatory reaction following mechanical injury of the chest appears similar to the autoimmune response following myocardial infarction in Dressler's syndrome. However, the examples of idiopathic pericarditis in immune-suppressed subjects, contradict the solely-lymphocyte-based theory (14).

Considering the current guidelines [1], finding the exact cause of the condition would not influence the therapeutic strategy in case of our patient. The pharmacological treatment is based on high doses of NSAIDs such as ibuprofen, naproxen and indomethacin. The suggested therapeutic duration depends on clinical course of the disease and range from 1–4 weeks for the first episode, to months when recurrent disease is present. Gradual dose reduction is indicated after a normal CRP level and echocardiographic image is restored. An additional medication is colchicine which, accumulating in the leukocytes, impairs the processes of chemotaxis, phagocytosis and degranulation thus preventing fibrosis and decreasing the rate of recurrence (15, 16). Administration of colchicine is indicated for 3 months following the acute episode in low doses (0.5–1 mg daily). When standard treatment is not effective, steroids should be administered, followed by azathioprine, intravenous immunoglobulin and anakinra in unresponsive cases (1, 17). Nevertheless, the drug therapy should be coupled with a complete refrain from physical activity.

Importantly, the medication that has played the greatest role in the treatment of our patient was prednisone which, due to its numerous side-effects (18) and an uncertain role in relapse-prevention, is listed among 2nd line drugs reserved for a specific group of patients and should only be administered in particular cases (Table 2), in the lowest effective dose [1]. Considering the risk of developing constrictive pericarditis in our patient, we decided that steroid-related side-effects were tolerable in this case.

**Table 2:** Indications for steroid therapy in pediatric pericarditis.

Contraindications to NSAIDs (renal failure)
No response to adequate treatment with NSAIDs
Recurrent pericarditis (combined with NSAIDs and colchicine)
A pericarditis in course of an autoimmune disease, that is routinely treated with steroids

The prognosis in pediatric pericarditis is generally favorable, with a complete remission in 85–90% of patients. Patients who require invasive treatment, such as pericardial drainage, are more likely to develop chronic pericarditis (17%) thus, they need longer observation and, in many cases repetitive treatment.

## Conclusions

In conclusion, although pericarditis is a rare cause of fever and chest pain in children, it poses a risk of recurrence and can lead to serious complications. Thus, patients with this condition require careful clinical and echocardiographic monitoring during, and after completing the treatment. A chest trauma should be taken into consideration when investigating the etiology of pericarditis.

## Data Availability Statement

The raw data supporting the conclusions of this article will be made available by the authors, without undue reservation.

## Ethics Statement

Written informed consent was obtained from the parents of the child for the publication of this case report.

## Author Contributions

PL and RS analyzed and interpreted the patient data regarding the pericarditis. PL performed the echocardiographic and electrocardiographic examination during the follow-up period and was a major contributor in writing the manuscript. Both authors read and approved the final manuscript.

## Conflict of Interest

The authors declare that the research was conducted in the absence of any commercial or financial relationships that could be construed as a potential conflict of interest.
