# Supplementary figures and images for: Case Report: Unexplained Fever and Chest Pain in a 5-Year-Old
Source: Front Pediatr. 2021 Jun 22;9:694678. doi: 10.3389/fped.2021.694678 (PMC8259459; doi:10.3389/fped.2021.694678)

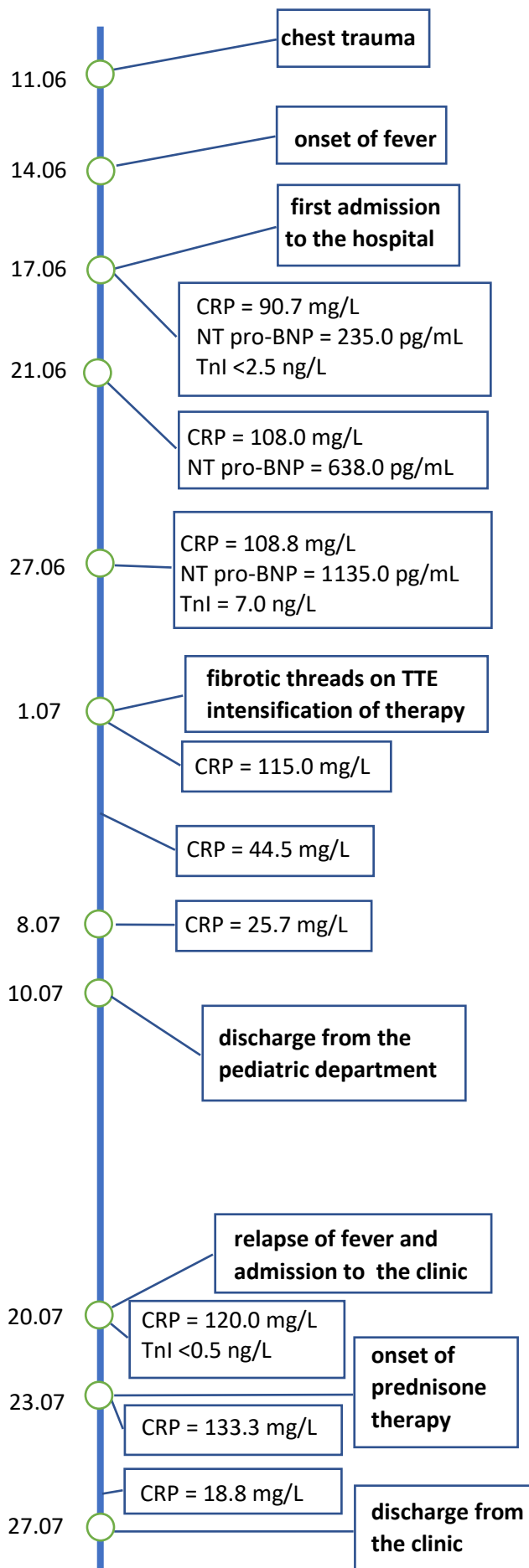

Supplement: Supplementary file 1 [file Data_Sheet_1.PDF]
